# Elucidation of pineapple softening based on cell wall polysaccharides degradation during storage

**DOI:** 10.3389/fpls.2024.1492575

**Published:** 2024-11-01

**Authors:** Fengjun Li, Xingzhou Xia, Lilang Li, Longlong Song, Yuping Ye, Yueming Jiang, Hai Liu

**Affiliations:** ^1^ College of Food Science and Technology, Guangdong Ocean University, Guangdong Provincial Key Laboratory of Aquatic Product Processing and Safety, Guangdong Provincial Science and Technology Innovation Center for Subtropical Fruit and Vegetable Processing, Guangdong Province Engineering Laboratory for Marine Biological Products, Key Laboratory of Advanced Processing of Aquatic Product of Guangdong Higher Education Institution, Zhanjiang, China; ^2^ Collaborative Innovation Center of Seafood Deep Processing, Dalian Polytechnic University, Dalian, China; ^3^ Guangdong Provincial Key Laboratory of Applied Botany, Key Laboratory of Postharvest Handling of Fruits of Ministry of Agriculture and Rural Affairs, South China Botanical Garden, Chinese Academy of Sciences, Guangzhou, China

**Keywords:** pineapple, fruit softening, cell wall, polysaccharide degradation, quality maintenance

## Abstract

The degradation of cell wall polysaccharides in pineapple fruit during softening was investigated in the present study. Two pectin fractions and two hemicellulose fractions were extracted from the cell wall materials of ‘Comte de Paris’ pineapple fruit at five softening stages, and their compositional changes were subsequently analyzed. The process of softening of the fruit corresponded to an increase in the water-soluble pectin (WSP) and 1 M KOH-soluble hemicellulose (HC1) fractions, and a decrease in the acid-soluble pectin (ASP) fraction, which suggested the solubilization and conversion of cellular wall components. However, the content of 4 M KOH-soluble hemicellulose (HC2) decreased and then returned to the initial level. Furthermore, WSP, ASP, and HC1 showed an increment in the content of low molecular weight polymers while a decline in the high molecular weight polymers throughout softening, and not significant change in the contents of different molecular polymers of HC2 was observed. Moreover, the galacturonic acid (GalA) content in the main chain of WSP was maintained at a relatively constant level, but the major branch monosaccharide galactose (Gal) in WSP decreased. Different from WSP, the molar percentages of Gal and GalA in ASP decreased. The Gal or Arabinose (Ara) in HC1 exhibited a gradual decline while the molar percentages of xylose (Xyl) and glucose (Glu) in the main chain increased. These suggested that the main chain of ASP degraded while the branched chains of ASP, WSP and HC1 depolymerized during pineapple softening. Overall, fruit softening of ‘Comte de Paris’ pineapple was found to be the result of differential modification of pectin and hemicellulose.

## 1 Introduction

Pineapple (*Ananas comosus* L.), a tropical and herbaceous monocot, is one of the most appreciated fruits worldwide due to its unique taste and flavor (Guillén et al., 2022). Postharvest softening improves in the texture, flavor, and aroma of the pineapple fruit to achieve an ideal edible state (Sun et al., 2013). Generally, pineapple fruit undergoes a gradual softening process during storage. However, excessive softening can lead to a reduction in the fruit’s edible quality and commercial value (Li et al., 2018). Therefore, elucidating the softening mechanism of pineapple fruit during storage is of great significance for the development of preservation technology.

The plant cell wall plays a decisive role in fruit firmness and exhibits a three-layered structure comprising a middle lamella, primary cell wall and secondary cell wall. The primary cell wall polysaccharides, which include pectin, hemicellulose, and cellulose, are cross-linked structures that maintain cell wall toughness and tissue integrity (Zhang et al., 2021). Therefore, polysaccharide degradation is a key factor to affect fruit texture and quality. The components of primary walls are modified during fruit ripening by the regulation of endogenous enzymes, including polygalacturonase, pectin methyl esterase, xyloglucan endotransglucosylase-hydrolase, expansin, endo-β-1,4-glucanase, and non-enzymatic factors, such as ·OH (Duan et al., 2011; Xu et al., 2022; Yang et al., 2021). The enzymatic and non-enzymatic disassembly of cell wall polysaccharides results in alterations to their molecular weight, monosaccharide composition, monosaccharide connectivity, and chain parameters (Lin et al., 2019; Prasanna et al., 2007). During fruit softening, the regulation of pectin primarily encompasses polymer modification (such as demethylation, deesterification, deacetylation, etc.), main chain breakage, branch chain loss, and molecular weight reduction (Sun et al., 2013). Accompanied with the degradation of pectin, hemicellulose polysaccharidesalso undergo constant modification, manifested by the main chain breakage, branch chain substitution and modification, and molecular weight reduction also occurred during fruit softening (Goulao and Oliveira, 2008; Ren et al., 2020). The modification of cellular wall polysaccharides in certain climacteric fruit have been reported, including tomato (Liu et al., 2023), banana (Duan et al., 2008), papaya (Manrique and Lajolo, 2004), peach (Brummell et al., 2004), apricot (Cui et al., 2021), plum (He et al., 2022) and custard apple (Ren et al., 2020). Meanwhile, information is now also available on the modification of cell wall polysaccharides in non-climacteric fruits, including sweet cherry (Salato et al., 2013), Chinese bayberry (Sun et al., 2013), blueberry (Wang et al., 2017), strawberry (Xu et al., 2022), and longan (Wang et al., 2013; Lin et al., 2023). These researches have revealed that numerous modification changes of cellular wall polysaccharides took place, such as solubilization and depolymerization of particular polymers, loss of certain neutral polysaccharides, broken of glycosyl linkages, were transient and occurred at different phases of the softening process. Variations in cellular wall compositions may result in differences in the chemical modifications associated with softening in each fruit species. However, except for banana, the above fruit belong to dicot plants, and it is generally believed that various changes in cell wall structure exist in fruit of dicots and monocots (Nothnagel and Nothnagel, 2007).

Preservation technology such as modified atmosphere packaging (MAP), wax coating, heat, CaCl_2_, and 1-methylcyclopropene (1-MCP) treatment were employed to prolong the storage time of pineapple fruit. Furthermore, these researches mainly focused on changes in physiological activity and quality during the softening process of pineapple (Li et al., 2018). However, there is a paucity of research investigating the in-depth mechanisms of pineapple fruit softening, particularly from a structural perspective. In this study, ‘Comte de Paris’ pineapple fruits were used to investigate the changes in cell wall polysaccharides during the softening process. The content, molecular weight, and monosaccharide composition were analyzed to determine the connection between the changes in cell wall polysaccharides and the fruit softening of pineapple. This work aimed to reveal the softening mechanism of pineapple fruit based on the degradation of cell wall polysaccharides and provide a theoretical basis for improving the storage and preservation methods of pineapple fruit.

## 2 Materials and methods

### 2.1 Plant materials

Fruit of pineapple (*Ananas comosus* cv. ‘Comte de Paris’) at an 80% maturity was harvested from a commercial orchard in Xuwen County, Zhanjiang City, Guangdong Province of China. Only fruit with uniform color and size without any damage were selected for the experiment. In the trials, five softening stages of pineapple fruit during storage were used. Stage I, 2 days after harvesting, >90% of skin green color; stage II, 4 days after harvesting, 80-90% of skin green color; stage III, 8 days after harvesting, 70-80% of skin yellow color; stage IV, 11 days after harvesting, 80-90% of skin yellow color; and stage V, 14 days after harvesting, > 90% of skin yellow color. The pineapple utilized in each ripening stage comprised 9 fruits, divided into three replicates. The pulp of the equatorial region of the fruit, excluding the skin and core, was used for the measurement of total soluble solids (TSS), titratable acid (TA), Vc, and firmness, while frozen in liquid nitrogen and stored at -80 ° for the extraction and analysis of cell walls.

### 2.2 Determinations of surface color and fruit firmness

The surface color of pineapple was determined according to the method outlined in our previous report (Li et al., 2021). For fruit firmness determination, a TMS-Pro food texture analyzer was used. The firmness of the flesh at 1 cm from the core was measured at the proximal, middle, and distal cross sections and the average value was taken. Six fruits were measured each time, and the firmness of fruit was expressed as Newtons (N).

### 2.3 pH and Vc

The method of Mir and Shahbazi (2022) was used to evaluate the pH value, with slight modifications. Pineapple flesh (10 g) was homogenized with 50 mL of distilled water for 1 min. After standing for 20 min, the pH of the homogenate was measured with an HI2210 pH meter. The measurements were repeated three times.

The Vc content of pineapple fruit was determined by the 2,6-dichlorophenol titration method (Wani and Uppaluri, 2022). A 20 g sample of pineapple flesh was ground with 100 mL of 2% oxalic acid, then 5 g of slurry was diluted to 50 ml with 2% oxalic acid solution and filtered to obtain a sample solution. A 10 mL of sample solution was taken and titrated with 0.1% 2,6-dichlorophenol, the consumed volume until a slight red color appeared and non-faded for 15 s was recorded, and the Vc content of pineapple fruit was determined by calculation with Vc standard solution.

### 2.4 TSS and TA

The determination of TSS content was conducted with a portable brix meter. Pineapple fruit (10g) was homogenized, then filtered with filter paper, and finally filtered juice was used for the determination. The results were expressed as a mass fraction (%). The TA content was measured by calculation with citric acid using the titration method (Bai et al., 2023).

### 2.5 Extraction of fruit cell wall material

The extraction of CWM was carried out according to the method of Vierhuis et al. (2000) with a slight modification. Pineapple pulp tissues (100 g) at different softening stages were placed into 300 mL of 95% (v/v) ethanol, homogenized, and then incubated 15 min in a boiling water bath to inactivate enzymes and destroy cell walls. After cooling, the homogenate was filtered while the residue was repeatedly washed and filtered with 70% ethanol until the filtrate exhibited no sugars, which was detected by the sulfuric acid phenol method. Then the insoluble residue was repeatedly extracted with chloroform/methanol (1:1, v/v), and then the extracted solution was filtered to remove the protein. The obtained component was subsequently stirred and washed with acetone to remove fats and pigments until the filtrate was colorless. Finally, 90% dimethyl sulfoxide was added to the filter residue for overnight extraction to remove starch, and 70% ethanol was then added for precipitation. The precipitate was collected and weighed as the cell wall materials (CWM) after drying to obtain a constant weight for 48 h at 30 °.

### 2.6 Extraction and determination of cell wall polysaccharides

The extraction of cell wall polysaccharides was carried out referring to the methods of Ren et al. (2020). CWM (0.8 g) was sequentially extracted with 50 mL of distilled water, 0.5 M HCl, 1 M KOH (containing 20 mM NaBH_4_), and 4 M KOH (containing 20 mM NaBH_4_) at 4 ° for 12 h by magnetic stirring. After each extraction, the solubilized polymers were separated from the insoluble residue by centrifugation at 8000 g for 20 min at 4 °. The extraction solution after centrifugation was obtained while the precipitate was collected and washed with 30 mL of distilled water, 0.5 M HCl, 1 M KOH, or 4 M KOH, respectively, and centrifuged again. The combined the two extraction solutions were then used as the water-soluble pectin (WSP), acid-soluble pectin (ASP), 1 M KOH-soluble hemicellulose (HC1), and 4 M KOH-soluble hemicellulose (HC2), respectively. Furthermore, the extraction was dialyzed with distilled water at 4 ° for 24 h (molecular weight retained by dialysis bag: 3500D). Prior to dialysis, the ASP extraction solution was adjusted to a neutral pH, whereas the HC1, HC2, and CWM residues were neutralized to a pH of 6.0 with acetic acid. After concentrating by rotary evaporation at 40 °, the contents of cell wall polysaccharides were freeze-dried and weighed, respectively.

### 2.7 Molecular weight distribution of cell wall polysaccharides

The molecular weight distributions of cell wall polysaccharides were conducted according to the previous method (Wang et al., 2013). The 6 mg mL^-1^ solution of cell wall polysaccharides was prepared using 0.1 M NaNO_3_ as a mobile phase, and then filtered with 0.45 µm microporous filter membrane before analysis. A high-performance liquid chromatography (LC-20A Shimadzu) equipped with a 7.8 × 300 mm chromatographic column (TSK-GEL G5000SWX) and differential refractive detector (RID-10A) was employed. The column temperature was 50 ° and 20 μL of samples were injected, with a flow rate of 1.0 mL min^-1^. A standard curve was constructed using the retention time of the dextran standard as the horizontal axis and the logarithm of the molecular weight (log Mw) of the dextran standard as the vertical axis. The average molecular weight of the sample was calculated according to the standard curve.

### 2.8 Analyses of monosaccharide composition of cell wall polysaccharides

The monosaccharide compositions of cell wall polysaccharides were analyzed by precolumn derivatization HPLC (Liu et al., 2021). Galacturonic acid (GalA), galactose (Gal), glucose (Glu), arabinose (Ara), xylose (Xyl), mannose (Man), rhamnose (Rha) standards and their mixed standards at 8 mM were prepared with ultrapure water. A sample of the cell wall polysaccharide (2.0 mg) was added to 2.0 mL of 2 M trifluoroacetic acid (TFA), filled with nitrogen, and hydrolyzed at 110 ° for 8 h. After cooling to room temperature, TFA was concentrated and evaporated under reduced pressure at 50 °, then 3 mL of methanol was added and evaporated to dryness. This process was repeated 4 times to remove the remaining TFA, then dissolved with ultrapure water and finally diluted to 1 mL. For derivatization, 500 μL of the mixed standard monosaccharide mother liquor and cell wall polysaccharide sample solution was added to 500 μL of 1-phenyl-3-methyl-5-pyrazolone (PMP)-methanol solution (0.5 M) and an equal volume of 0.3 M NaOH solution. The mixture was reacted in a water bath at 70 ° for 30 min and then neutralized with 500 μL of 0.3 M HCl. Subsequently, 1 mL chloroform was added, then extracted three times, and finally the upper liquid was obtained as the derivatized mother liquor. Filtering through a 0.45 μm filter and dilution in the mother liquorto 4, 2, and 1 mM gradient solutions was conducted before injection. The chromatographic conditions were as follows: a HPLC (LC-20A Shimadzu) with a 250 × 4.6 mm chromatographic column (Shim-pack VP-ODS) and UV detector at 250nm. Mobile phase consisted of 0.05 M phosphoric acid buffer (KH_2_PO4-NaOH, pH 6.2) containing 7% acetonitrile. The injection volume of 10 μL and the flow rate of 1 mL min^-1^ were used, and the column temperature was 25 °.

### 2.9 Statistical analysis

All assays in this study were performed in triplicate. All data were presented as the mean ± standard error (SE) and then analyzed by one-way ANOVA to test for significant differences usingDuncan’s test (*P* < 0.05). Results were visualized with the SigmaPlot 10.0 software. Pearson correlation coefficients were performed using Origin 2021 software.

## 3 Results

### 3.1 Changes in visual appearance during pineapple fruit softening

**Figure 1:**
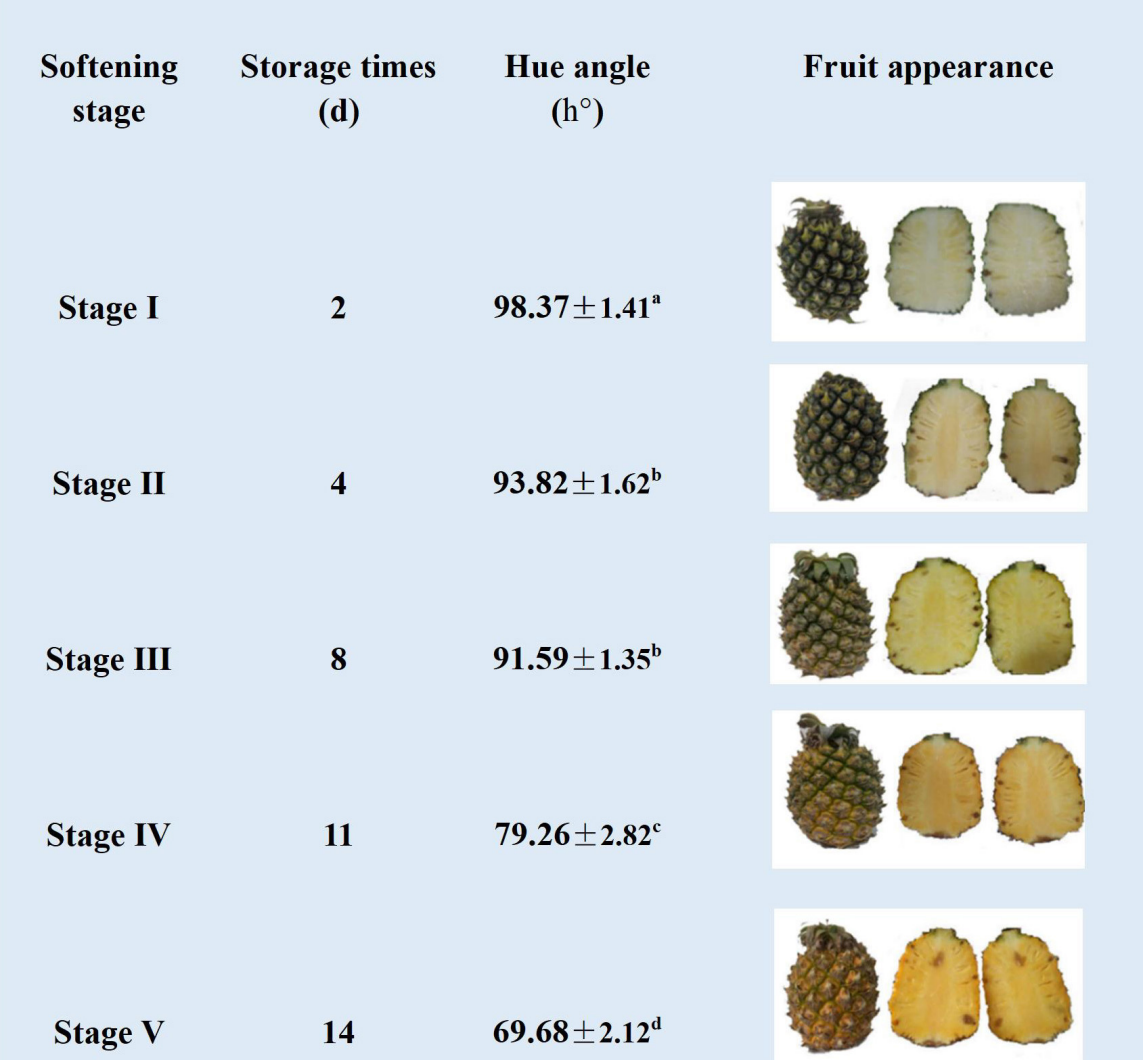
Changes in hue angle h° and visual images (surface and cross-sectional images) of different softening stages of pineapple fruit during storage at 25 ° (I: dark green, II: green, III: greenish yellow, IV: yellowish green, V: yellow). Each data point represented the mean ± SE of three replicates.

As showed in **Figure 1**, during the storage period (1–14 d), the fruit exhibited a green coloration initially, which gradually transitioned to a greenish yellow (stage III), then to yellowish green (IV), and ultimately to yellow at the end of storage. In agreement with color change, the hue angle h° of pineapple fruit remained unchanged during the initial storage, and then decreased rapidly from 91.59° (stage III) to 79.26° (stages IV). Additionally, the flesh of the fruit exhibited a change in color from light yellow (stages I and II) to yellow (stage III) and finally to orange-yellow (stages IV and V).

### 3.2 Changes in pH, Vc, TSS, and TA during pineapple fruit softening

**Figure 2:**
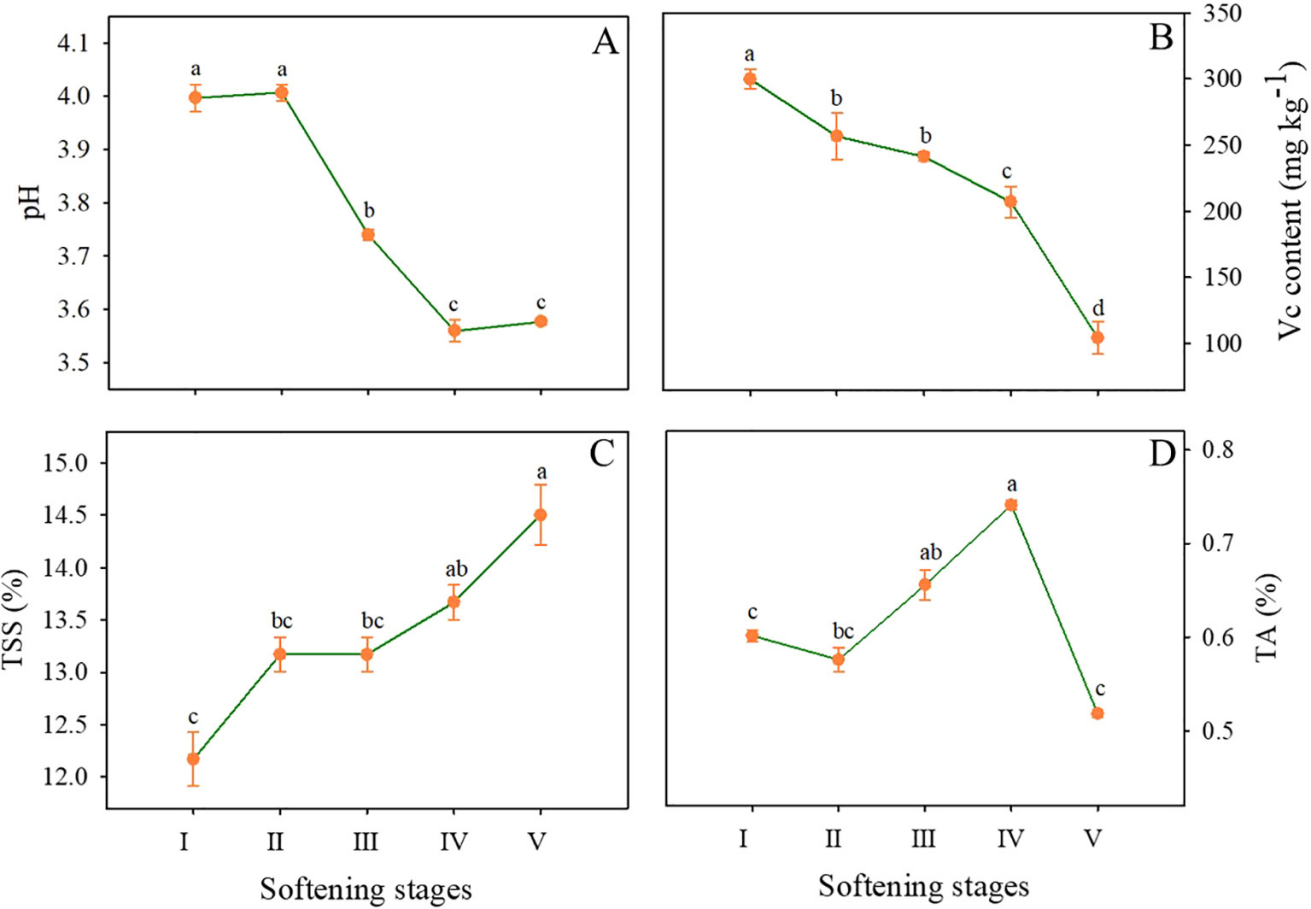
Changes in pH **(A)**, Vc **(B)**, TSS **(C)**, and TA **(D)** contents in the flesh of pineapple fruit at various softening stages. Each data point represented the mean ± SE of three replicates.

As shown in **Figure 2A**, the pH of the pineapple remained unchanged from stage I to II, decreased rapidly from 4.00 (stage II) to 3.47 (stage III), continued to decline, and reached the minimum value of 3.5 at stage IV. The content of Vc decreased rapidly throughout the storage and dropped to one-third of the initial level by the end of storage (**Figure 2B**). Fruit quality and flavor are directly related to TSS and TA. Generally, the TSS content presented an upward trend throughout the storage, especially at stages IV and V, reaching 13.67% and 14.5%, respectively (**Figure 2C**). As illustrated in **Figure 2D**, the TA content exhibited a slight decline from stage I to II, followed by an increase until stage IV, reached a maximum value of 14.5%. However, a notable decline was observed at the final stage of storage.

### 3.3 Changes in firmness and content of cell wall material (CWM) during pineapple fruit softening

**Figure 3:**
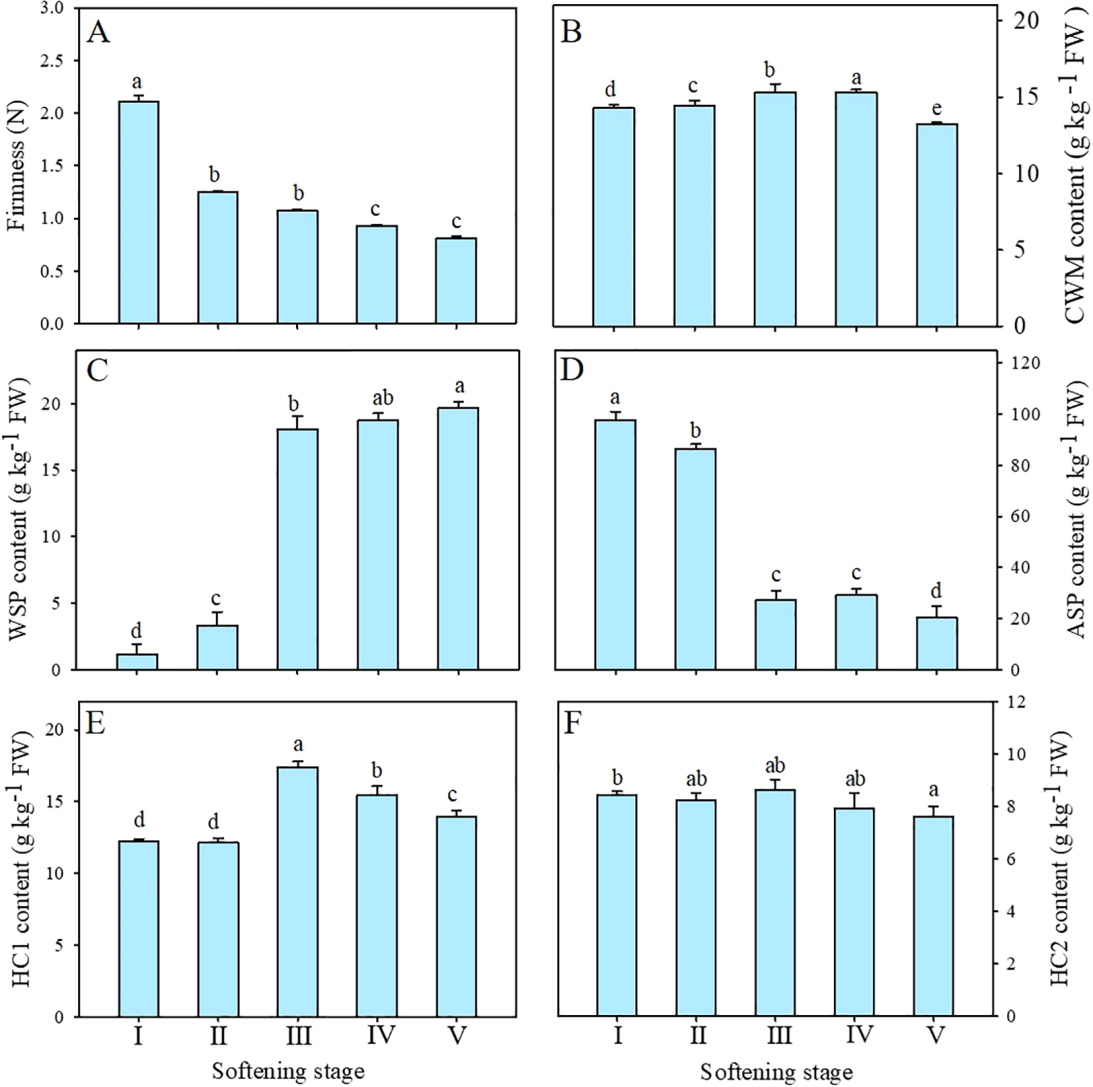
Changes in firmness **(A)**, CWM **(B)**, WSP **(C)**, ASP **(D)**, HC1 **(E)**, and HC2 **(F)** contents of pineapple fruit during softening. CWM, cell wall material; WSP, water-soluble pectin; ASP, acid-soluble pectin; HC1, 1 M KOH-soluble hemicellulose; HC2, 4 M KOH-soluble hemicellulose. Each data point represented the mean ± SE of three replicates.

The flesh becomes soft and juicy as indicated by the softening of ‘Comte de Paris’ pineapple fruit during storage. As demonstrated in **Figure 3A**, the firmness of the pineapple exhibited a rapid decline from stage I to II, followed by a slight decrease. By stage V, the firmness of the pineapple had decreased to the lowest level, only 38.86% of the value at the beginning of storage.

CWM was generally used in the study of cell wall components. As shown in **Figure 3B**, the content of CWM maintained a relatively stable at the preliminary stage but exhibited a notable decline at stage V, revealing that the proportion of cell wall polysaccharides decreased with the enhanced softening of pineapple.

### 3.4 Changes in pectin and hemicellulose polysaccharides during pineapple fruit softening

**Figure 4:**
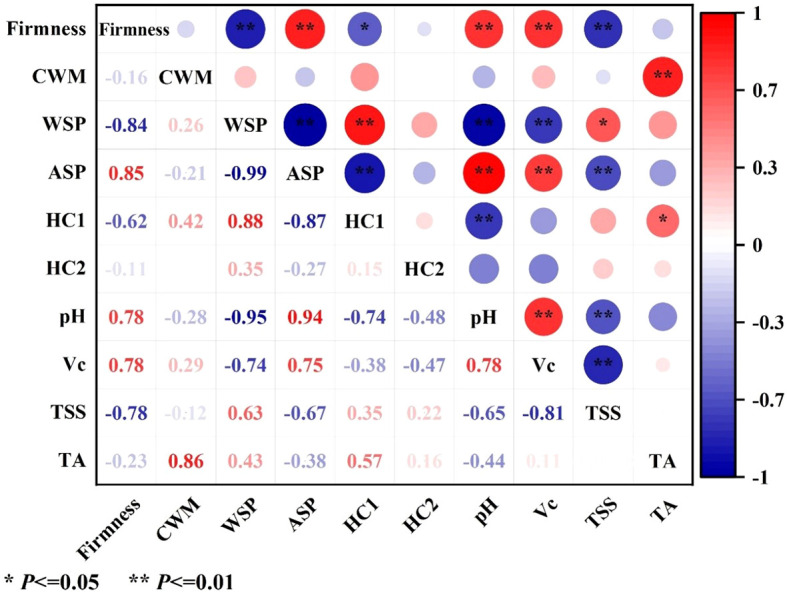
Correlation analyses of firmness, cell wall material (CWM), cell wall polysaccharides (WSP, ASP, HC1, and HC2), pH, Vc, TSS, and TA in pineapple fruit. CWM, cell wall material; WSP, water-soluble pectin; ASP, acid-soluble pectin; HC1, 1 M KOH-soluble hemicellulose; HC2, 4 M KOH-soluble hemicellulose. Red indicates positive correlation, blue indicates negative correlation, with larger circles and darker colors representing stronger correlations. *Significant at the 0.05 level; **Significant at the 0.01 level.

Among these cell wall polysaccharides, both WSP and ASP belonged to pectin polysaccharides. As shown in **Figure 3C**, the content of WSP of pineapple fruit during softening showed a significant increase from 3.30 g kg^-1^ at stage II to 18.05 g kg^-1^ at stage III and then maintained a moderate upward trend. Conversely, the content of ASP significantly decreased from 86.55 g kg^-1^ at stage II to 227.18 g kg^-1^ at stage III (**Figure 3D**) and then decreased gradually. Correlation analysis showed that WSP was significantly negatively correlated with firmness (-0.85, P < 0.01), whereas ASP showed a positive correlation (0.84, P < 0.01) (**Figure 4**).

HC1 and HC2 as hemicelluloses are loosely and tightly connected to cell walls, respectively. As shown in **Figure 3E**, the initial content of HC1 was 12.25 g kg^-1^ and exhibited no significant change until stage II, then increased significantly at stage III and finally increased to 13.96 g kg^-1^ at stage V. The content of HC2 exhibited a slight decline at the outset of the storage, followed by a return to approximately equal levels at stages IV and V. (**Figure 3F**). Correlation analysis exhibited that HC1 (-0.62, P < 0.05) but not HC2 (-0.11) was negatively correlated with fruit firmness of pineapple (**Figure 4**).

### 3.5 Changes in molecular weight distribution of cell wall polysaccharides during pineapple fruit softening

As shown in the HPLC analysis, the retention times of the standard dextrans with molecular weights of 10, 40, 70, 500, and 2000 kD were determined to be 9.81, 9.11, 8.69, 7.79, and 7.03 min, respectively. Thus, the regression equation log Mw =−0.8322 t + 9.1512 (r^2^ = 0.9977) was established based on the linear relationship between the retention time of standard dextrans and the logarithm of their molecular weight (**Supplementary Figure S1**) to analyze the molecular weight distributions of WSP, ASP, HC1 and HC2 at stages I, III and V.

#### 3.5.1 Molecular weight distributions of WSP and ASP during pineapple fruit softening

**Figure 5:**
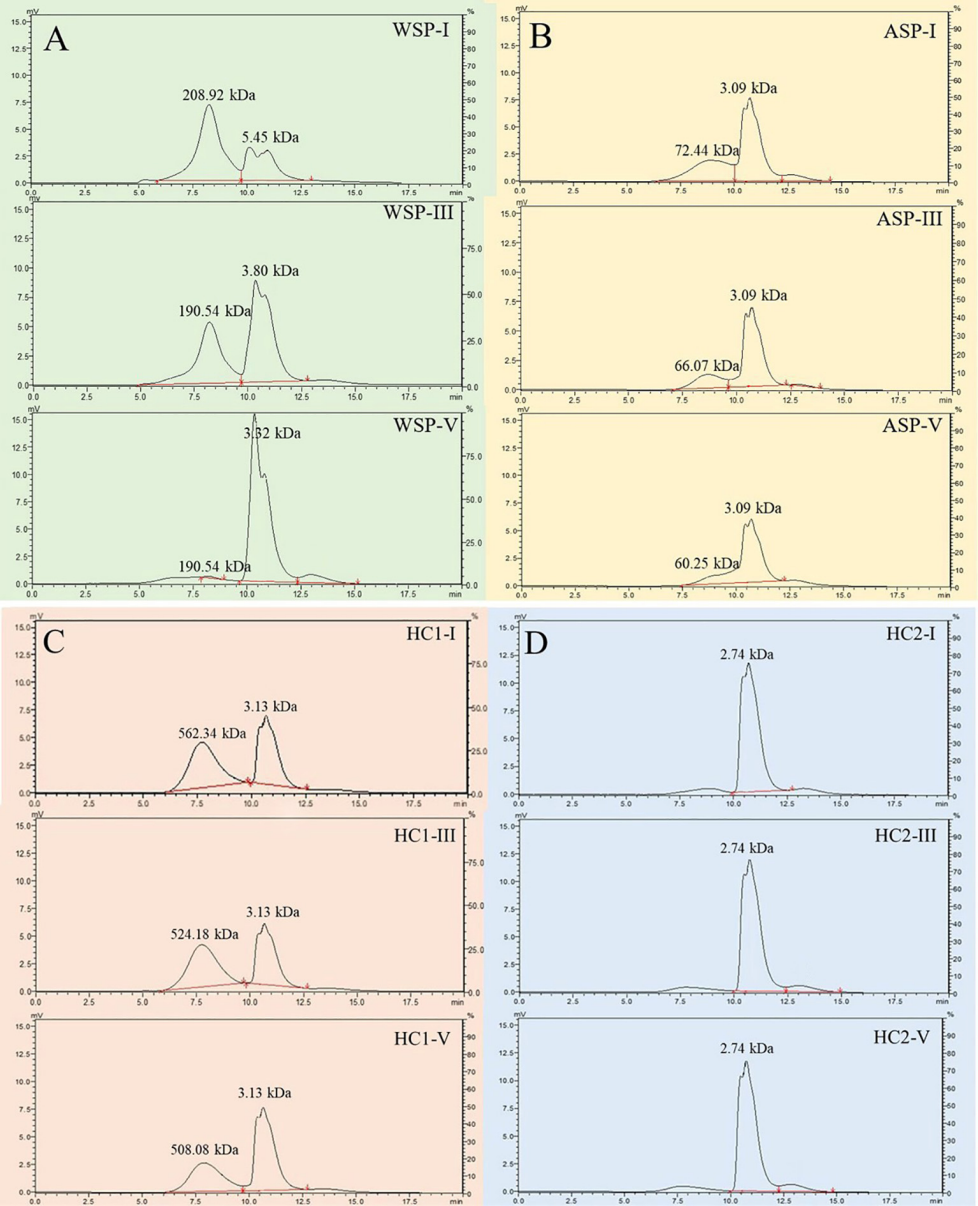
Molecular weight distributions of pectin polysaccharides (**(A)** WSP of I, III, V; **(B)** ASP of I, III, V) and hemicelluloses (**(C)** HC1 of I, III, V; **(D)** HC2 of I, III, V) in pineapple fruit at various softening stages. WSP, water-soluble pectin; ASP, acid-soluble pectin; HC1, 1 M KOH-soluble hemicellulose; HC2, 4 M KOH-soluble hemicellulose.

As shown in **Figure 5A**, two major elute peaks appeared in the WSP fraction, one with a high molecular weight of about 200.00 kDa, and the other with a low molecular weight of less than 10 kDa. With fruit softening of pineapple, the polymers with high molecular weight in WSP showed a slight change, from 208.92 kDa at stage I to 190.54 kDa at stage III, and finally to 193.00 kDa. The size of low molecular weight polymers also did not change significantly. However, the WSP fraction with higher molecular weight showed atrend of conversion toward WSP fraction with lower molecular weight during fruit softening. Concerning ASP, there were two elute peaks with different molecular weights, one with a larger molecular weight of about 70.00 kDa and the other with a lower molecular weight at 3.09 kDa (**Figure 5B**). Concomitantly, during the softening of pineapple fruit, ASP underwent a depolymerization, which showed an increment in the content of the low molecular weight fractions while the decline in the high molecular weight fractions. Distinct from the WSP, the ASP fraction with high molecular weight gradually decreased from 72.44 kDa at stage I to 60.25 kDa at stage III during fruit softening.

#### 3.5.2 Molecular weight distributions of HC1 and HC2 during pineapple fruit softening

The fraction with high molecular weight exhibited a slight alteration, deceasing from 562.34 kDa at stage I to 508.08 kDa at stage III, while the one with low molecular weight maintained 3.13 kDa. Meanwhile, its content demonstrated a decline tendency during fruit softening of pineapple (**Figure 5C**). The content conversion between HC1 with high and low molecular weights was significantly less than that of WSP, indicating that HC1 underwent less extensive depolymerization. As illustrated in **Figure 5D**, the eluted peak of HC2 maintained an average molecular weight of 2.74 kDa and demonstrated minimal fluctuations in content with fruit softening.

### 3.6 Changes in monosaccharide compositions of cell wall polysaccharides during pineapple fruit softening

**Supplementary Figure S2** exhibited the retention time of the dextran standards. Based on the linear regression in **Supplementary Table S1**, the monosaccharide composition of each cell wall polysaccharide was estimated.

#### 3.6.1 Changes in monosaccharide compositions of WSP and ASP during pineapple fruit softening

**Figure 6:**
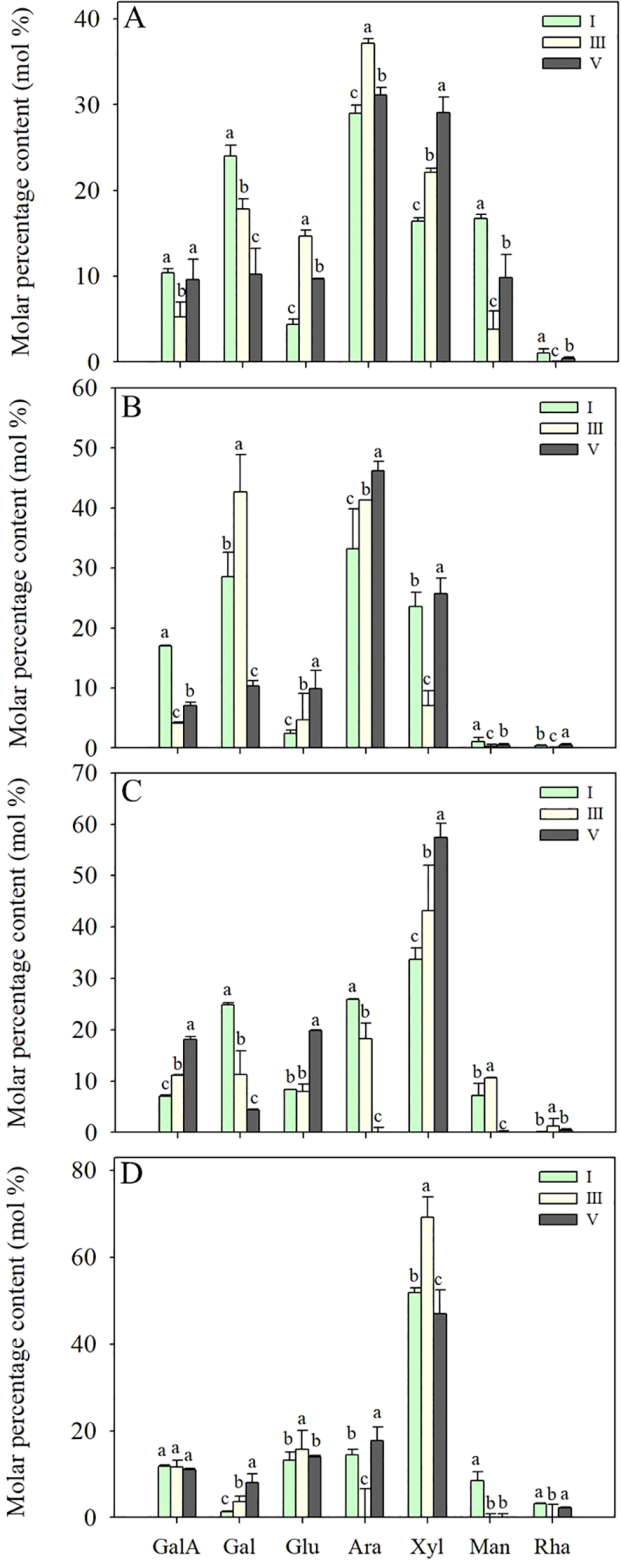
Monosaccharide compositions of WSP **(A)**, ASP **(B)**, HC1 **(C)** and HC2 **(D)** polysaccharide fractions from flesh tissues of ‘Comte de Paris’ pineapple fruit at various softening stages. Each data point represented the mean ± SE of three replicates. WSP, water-soluble pectin; ASP, acid-soluble pectin; HC1, 1 M KOH-soluble hemicellulose; HC2, 4 M KOH-soluble hemicellulose; GalA, galacturonic acid; Gal, galactose; Glu, glucose; Ara, arabinose; Xyl, xylose; Man, mannose; Rha, rhamnose.

As shown in **Figures 6A, B**, the WSP and ASP in cell walls contained a large number of neutral sugar components, such as arabinose (Ara), galactose (Gal), and xylose (Xyl), accounting for about 70% of the total monosaccharide compositions. Additionally, galacturonic acid (GalA), glucose (Glu), mannose (Man), and rhamnose (Rha) were detected. During fruit softening, the relative content of Gal in the WSP significantly decreased from 23.97 to 10.20%, whereas the molar percentage of GalA remained relatively stable. The molar percentages of Gal and Ara in ASP were 19.2% and 16.5% higher than WSP, respectively, indicating that the ASP had richer branched chains. During fruit softening of pineapple, there was a notable decline in the molar percentage of GalA in the ASP, while the relative Gal content increased from stage I to III and then decreased to a minimum of 10.32% at stage V.

#### 3.6.2 Changes in monosaccharide compositions of HC1 and HC2 during pineapple fruit softening

The characteristic monosaccharide of hemicellulose, Xyl, was richer in HC1 and HC2 than those pectin fractions, followed by Glu, Ara, Gal, GalA, and a minor amount of Man and Rha (**Figures 6C, D**). During pineapple softening, the molar percentages of Xyl and Glu in the main chain showed an increasing tendency from 33.73% and 8.31% at stage I to 57.43% and 19.87% at stage V, respectively. In comparison, the molar percentages of Gal and Ara in HC1 gradually reduced from 24.82 and 25.93% at stage I to 4.36 and 23.41% at stage V. Additionally, it was also observed that the percentages of Gal in HC2 gradually increased, while the percentages of other monosaccharides did not change significantly.

## 4 Discussion

Despite extensive research having been carried out on the softening of various fruit, there is a paucity of information available regarding monocots. Pineapple is a tropical monocotyledonous fruit, and the proper softening is important for the formation of its edible quality. However, little information is available on fruit softening in pineapple. In this study, ‘Comte de Paris’ pineapple fruits were used to investigate the changes in cell wall polysaccharides during the softening process.

During pineapple softening, the surface color and firmness decreased (**Figures 1**, **3A**) while TSS and TA increased (**Figures 2C, D**), which was consistent with the better texture and richer flavor. However, excessive softening would reduce the commodity quality, and reduce the desire to consume. For example, Vc as an important indicator of the nutritional quality of fruits, decreased with pineapple softening (**Figure 2B**). In addition to cellular wall degradation, the pH level is noted in controlling the process of cell wall relaxation, as it can affect the activities of cell wallmodifying enzymes during softening. Studies have shown that the polygalacturonase in banana fruits was most active at pH 3.5, but its activity gradually declined as the pH increased from 4.0 to 6.0 (Gayathri and Nair, 2014). As shown in **Figure 2A**, the pH of ‘Comte de Paris’ pineapple also decreased with the fruit softening, which can provide a guarantee for the high activities of cell wall-modifying enzymes of pineapple fruit and in turn lead to cell wall degradation and fruit softening. In the present study, cell wall materials (CWM) were extracted to clarify the softening pattern of the ‘Comte de Paris’ pineapple. The result showed that the content of CWM slightly increased at the initial stage, but then significantly decreased subsequently (**Figure 3B**), revealing that the proportion of cell wall polysaccharides decreased with the excessive fruit softening.

Structural alterations in cell wall polysaccharides were assumed to be the basis for the cell wall degradation and subsequent fruit softening (Paniagua et al., 2014). During fruit softening, pectin polysaccharides were gradually converted from insoluble protopectin to soluble pectin and pectin acid, and the adhesion between adjacent cells was reduced (Wang et al., 2018). The WSP consisting mainly of soluble pectin, pectinic acid, and pectinate salts loosely connects to cell walls, while ASP is mainly composed of insoluble protopectin to tightly bind to the cell walls (Duan et al., 2008; Wang et al., 2021). In the present study, with enhanced softening of pineapple fruit, the WSP content gradually increased, while the ASP content significantly decreased, indicating that the pectin polysaccharide was continuously dissolved and transitioned from insoluble to soluble forms (**Figures 3C, D**). Previous research have shown that the WSP content gradually increased during ripening and softening of banana, papaya, longan, apricot, plum, and tomato, and a significant correlation between pectin dissolution and the reduced fruit firmness was observed (Bu et al., 2013; Duan et al., 2008; Manrique and Lajolo, 2004; Cui et al., 2021; He et al., 2022; Lin et al., 2023; Yun et al., 2022). In this study, HC1 and HC2 were extracted by 1 M and 4 M KOH solutions, respectively, accounting for loosely and tightly bound hemicellulose. **Figure 3E** showed that the content of HC1 increased substantially while the content of HC2 slightly decreased, but they returned to the initial level (**Figure 3F**). Based on the changes in the HC1 and HC2 contents, it was speculated that tightly-combined matrix polysaccharides were degraded and then converted into loosely-combined matrix polysaccharides during the softening of pineapple, which was observed also in the ripening of custard apple and Chinese bayberry fruit (Ren et al., 2020; Sun et al., 2013). Correlation analysis further showed that HC1 but not HC2 was negatively correlated with fruit firmness (**Figure 4**), suggesting that besides pectin, hemicellulose also plays an important role in the softening of pineapple fruit.

In general, the various molecular weight distributions and neutral monosaccharide compositions of cell wall polysaccharides are considered to have great impact on the structure of cell wall polysaccharides (Salato et al., 2013). As shown in **Figure 5**, it was observed that the various molecular weight distributions of WSP, ASP, and HC1 exhibited the occurrence of depolymerization to different extents. The contents of WSP and HC1 fractions with high molecular weight tended to decrease gradually, while their fractions with low molecular weight increased during fruit softening. Distinct from WSP and HC1, the depolymerization of ASP not only embodied in the content alternation, but the ASP fraction with high molecular weight showed a downshift during fruit softening. The change in the molecular weight distribution of these cell wall polysaccharides in pineapple is not consistent with banana, another reported monocot, which has undergone changes in the molecular weight of not only WSP, ASP, and HC1, but also HC2 (Duan et al., 2008; Cheng et al., 2009). Pectin polysaccharides are generally composed of a homogalacturonan (HG) backbone, with rhamnogalacturonan-I (RG-I) and rhamnose residues interspersed in HG gaps (Liu et al., 2023). Galactose and arabinose are generally present in the branched chain of the pectin polysaccharides in the form of glycosides, and the loss of galactose and/or arabinose is thought to be a common feature of fruit softening (Gross and Sams, 1984). During the softening of pineapple fruit, the GalA content in the main chain of WSP maintained basically, but the proportion of Gal in WSP significantly decreased (**Figure 6A**), suggesting that the main chain did not depolymerize but other branched chains of WSP depolymerized, resulting in the loss of Gal. Different from WSP, the molar percentages of Gal and GalA in ASP decreased during pineapple softening (**Figure 6B**), indicating that both the main and branched chains of ASP were broken and degraded, leading to reduced GalA and Gal.

Hemicelluloses as the primary cell walls connect to celluloses by hydrogen bonds and, thus, play a very important role in maintaining the shape of cells and the mechanical strength of plant tissue (Scheller and Ulvskov, 2010). Hemicelluloses belong to a group of heterogeneous polysaccharides, whose molecular chain is composed of sugar units, including xyloglucans, xylans, mannans, glucomannans and β-(1→3,1→4)-glucans. The monosaccharide compositions of HC1 and HC2 both displayed a high amount of Xyl, followed by Glu, Ara, Gal, GalA, and a small amount of Man or Rha. The monosaccharide composition of pineapple hemicellulose in the present study is consistent with previous reports by Smith and Harris (1995). Intriguingly, the HC1 and HC2 in banana flesh are rich in Glu, Xyl, and Man (Cheng et al., 2009), which differs from the composition of pineapple. In the present study, the amount (mol %) of Gal or Ara in HC1 tended to decrease gradually while the molar percentages of Xyl and Glu in the main chain increased during fruit softening (**Figure 6C**), suggesting that the pineapple hemicellulose was mainly composed of xyloglucan and arabinogalactan polysaccharide. Furthermore, it was suggested that releases of neutral sugars, such as Gal and Ara from branch chains in the HC1, was closely related to the softening of pineapple fruit. During the softening, the molar percentage of Gal in HC2 gradually increased and that of Man decreased, while the other monosaccharides did not change significantly (**Figure 5D**). The changes in the monosaccharide composition of HC1 and HC2 are consistent with a previous report in peach fruit where disaggregation occurred only in loosely connected components (Brummell et al., 2004).

## 5 Conclusions

**Figure 7:**
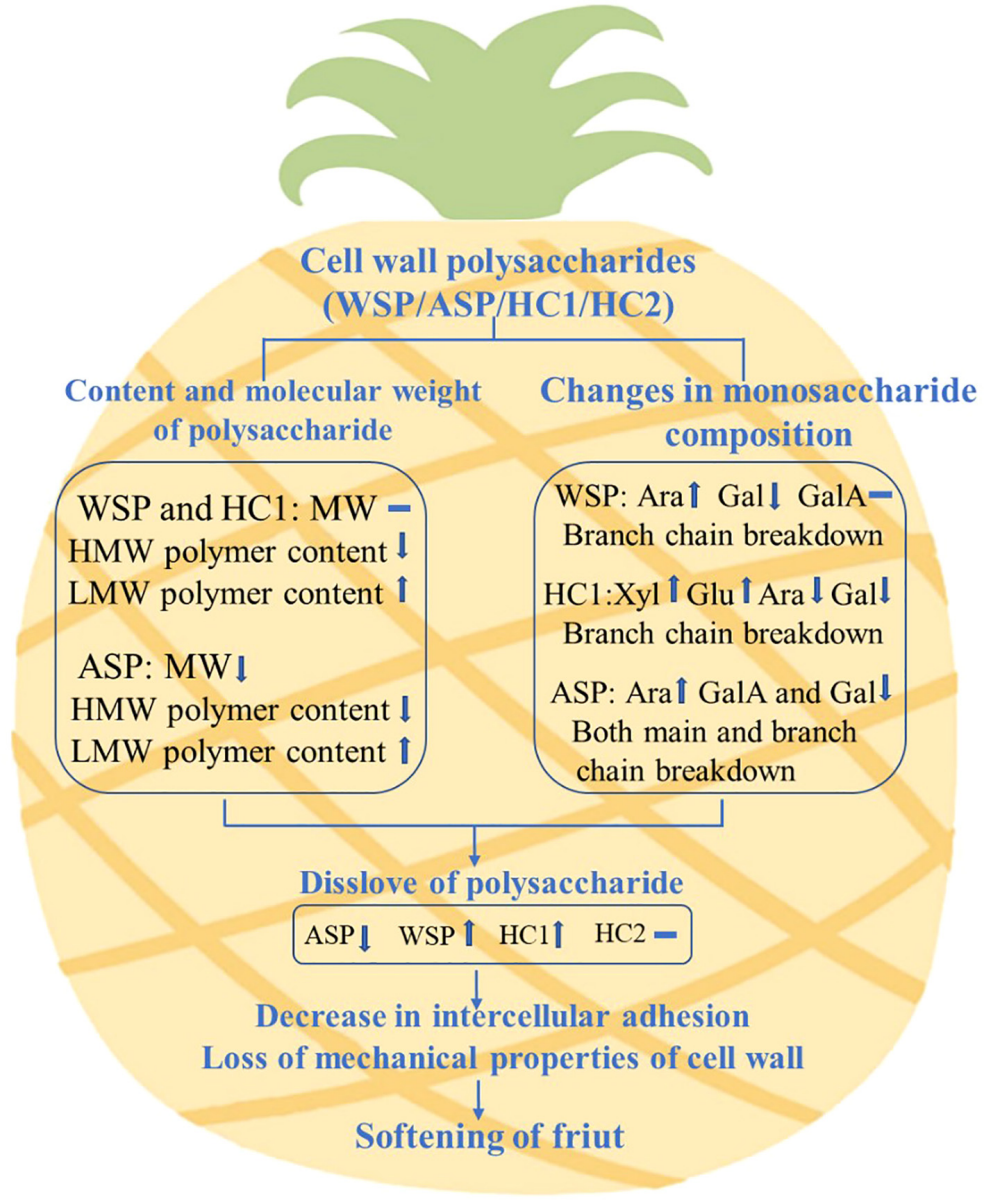
Possible mechanism of pineapple fruit softening caused by the degradation of cell wall polysaccharides. MW indicates molecular weight, HMW indicates high molecular weight, LMW indicates low molecular weight.

In conclusion, the present study demonstrated that the content, molecular weight distribution and monosaccharide compositions of WSP, ASP, and HC1 changed greatly during pineapple fruit softening, and WSP, ASP, HC1 but not HC2 were correlated with fruit firmness, which suggested the involvement in the softening process of ‘Comte de Paris’ pineapple fruit. The possible mechanism of pineapple fruit softening caused by the degradation of cell wall polysaccharides was proposed, as shown in **Figure 7**. The present study can provide insight into the role of the degradation of cellular wall polysaccharides in fruit softening, which is crucial for understanding the formation and deterioration of pineapple quality, and contribute to further technology for maintaining or preserving pineapple. To gain a better understanding of the degradation model of pineapple cell wall polysaccharides, further analysis of glycosidic bonds is essential. Additionally, it is crucial to investigate the impact of degradation-inducing factors, including both enzymatic and non-enzymatic processes, on these polysaccharides.

## Data Availability

The original contributions presented in the study are included in the article/**Supplementary Material**. Further inquiries can be directed to the corresponding author/s.
