# Supplementary material for: Elucidation of pineapple softening based on cell wall polysaccharides degradation during storage
Source: Front Plant Sci. 2024 Nov 1;15:1492575. doi: 10.3389/fpls.2024.1492575 (PMC11574306; doi:10.3389/fpls.2024.1492575)
Supplement: Supplementary file 1 [file DataSheet1.docx]

Supplementary Material

**Table S1** Standard curve of standard 1-phenyl-3-methyl-5-pyrazolone (PMP)-sugars

| Monosaccharide standards | Retention time (min) | Regression equation | Correlation coefficient |
| --- | --- | --- | --- |
| GalA | 4.729 | Y=85412.67X-24960.74 | R=0.992 |
| Gal | 7.197 | Y =101045.2X+38101.4 | R=0.996 |
| Glu | 8.866 | Y=196215.4X+13636.75 | R=0.992 |
| Ara | 9.763 | Y=51354.38X+42027.85 | R=0.993 |
| Xyl | 11.351 | Y =88413.64X+98826.64 | R=0.997 |
| Man | 12.932 | Y =244706.4X+13190.71 | R=0.991 |
| Rha | 25.098 | Y = 237785.6X-46675.74 | R=0.999 |


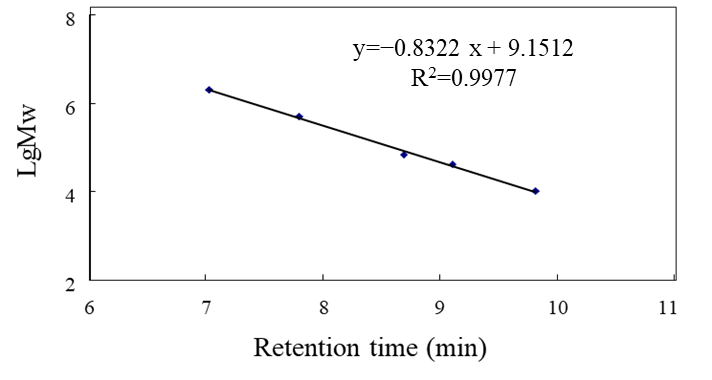


**Supplementary Figure 1.** The standard curve according to the retention time of standard dextrans and the logarithm of their molecular weight.

0.0

5.0

10.0

15.0

20.0

25.0

30.0

min

0

25

50

75

100

125

mV

1

2

3

4

5

6

7

**Supplementary Figure 2.** The HPLC separations of standard PMP-sugars (8 mmol L^-1^).

1, Galacturonic acid (GalA); 2, Galactose (Gal); 3, Glucose (Glu); 4, Arabinose (Ara); 5, Xylose (Xyl); 6, Mannose (Man); and 7, Rhamnose (Rha)
